# Josicélia Dumêt Fernandes’ professional trajectory: contributions to psychiatric and mental health nursing

**DOI:** 10.1590/0034-7167-2023-0174

**Published:** 2024-05-03

**Authors:** Ingredy Nayara Chiacchio Silva, Gilberto Tadeu Reis da Silva, Itanna Vytoria Sousa Serra, Deybson Borba de Almeida, Giselle Alves da Silva Teixeira, Rosana Maria de Oliveira Silva, Ana Lúcia Arcanjo Oliveira Cordeiro

**Affiliations:** I Universidade Federal da Bahia. Salvador, Bahia, Brazil; II Universidade Estadual de Feira de Santana. Feira de Santana, Bahia, Brazil

**Keywords:** Nursing, History of Nursing, Biography, Psychiatric Nursing, Mental Health, Enfermería, Historia de la Enfermería, Biografía, Enfermería Psiquiátrica, Salud Mental, Enfermagem, História da Enfermagem, Biografia, Enfermagem Psiquiátrica, Saúde Mental

## Abstract

**Objectives:**

to analyze nurse Josicélia Dumêt Fernandes’ life story, with emphasis on her work in the psychiatry and mental health fields.

**Methods:**

historical, qualitative research. Semi-structured interviews and documentary research were used as data collection techniques, collected from September to October 2021. For data analysis, we opted for the content analysis method and comparison with the Foucauldian philosophical framework.

**Results:**

four categories emerged: Transforming herself and mental health practices; (Re)framing professional practice; Nursing practice and power relations; and The paths and implications in the psychiatry and mental health fields.

**Final Considerations:**

the study of the biographer demonstrates a search for transformation of herself and mental health practices, with a rupture in paradigms and reframing of her practice in psychiatry and mental health.

## RESUMO

**Objetivos:**

analisar a história de vida da enfermeira Josicélia Dumêt Fernandes, com ênfase em sua atuação na área da psiquiatria e saúde mental.

**Métodos:**

pesquisa histórica, do tipo qualitativa. Foram utilizadas como técnicas de coleta de dados a entrevista semiestruturada e a pesquisa documental, coletados de setembro a outubro de 2021. Para a análise dos dados, optou-se pelo método de análise de conteúdo e a confrontação com o referencial filosófico foucaultiano.

**Resultados:**

emergiram quatro categorias: A transformação de si e das práticas em saúde mental; A (re)significação da prática profissional; A prática de enfermagem e as relações de poder; e Os caminhos e implicações no campo da psiquiatria e da saúde mental.

**Considerações Finais:**

o estudo da biografada demonstra uma busca por transformação de si e das práticas de saúde mental, com ruptura de paradigmas e ressignificações da sua prática em psiquiatria e saúde mental.

## RESUMEN

**Objetivos:**

analizar la historia de vida de la enfermera Josicélia Dumêt Fernandes, con énfasis en su trabajo en el área de psiquiatría y salud mental.

**Métodos:**

investigación histórica, cualitativa. Se utilizaron como técnicas de recolección de datos entrevistas semiestructuradas e investigación documental, recolectados de septiembre a octubre de 2021. Para el análisis de los datos, se optó por el método de análisis de contenido y comparación con el marco filosófico foucaultiano.

**Resultados:**

surgieron cuatro categorías: Transformación del yo y de las prácticas de salud mental; El (re)significado de la práctica profesional; Práctica de enfermería y relaciones de poder; y Los caminos e implicaciones en el campo de la psiquiatría y la salud mental.

**Consideraciones Finales:**

el estudio de la biógrafa demuestra una búsqueda de transformación de ella misma y de las prácticas de salud mental, rompiendo paradigmas y resignificando su práctica en psiquiatría y salud mental.

## INTRODUCTION

This article deals with Bahian nurse and professor Josicélia Dumêt Fernandes’ life story, from the perspective of her work in the psychiatry and mental health fields. Her life trajectory was linked to the defense of quality teaching, marked by her commitment to training critical and reflective nurses, capable of dealing with the demands and challenges of the profession, and she worked in mental health services, always seeking to guarantee optimal care humanized and quality care for patients.

This study arose from the approach of one of the authors, when developing their master’s thesis on the subject of nursing management in the mental health service of the *Hospital Universitário Professor Edgard Santos* (HUPES) - the first general hospital in Brazil to implement the Psychiatry Service, when they observed a gap in the study site’s history, and in the history of the professionals who contributed to the care provided by the sector over time. However, in informal reports on the topic, the contribution of nurse and professor Josicélia Dumêt Fernandes to strengthening the Mental Health service at HUPES, as well as in the process of Psychiatric Reform, was a recognized fact, motivating the construction of this study.

She graduated in nursing in 1965 at *Universidade Federal da Bahia* School of Nursing (EEUFBA). She worked as a nursing coordinator at *Casa de Saúde Ana Nery* as a nurse on duty, head of nursing at the HUPES psychiatric clinic, between 1960 and 1980. She completed her master’s degree in public health in 1984 and her doctoral degree in nursing in 1990. In 1992, she worked as chief of staff at the UFBA rectory. Between 1993 and 1999, she maintained her employment at UFBA, first as a visiting professor (until 1997) and then as a collaborating professor in the Graduate Program in Nursing, through the Support Program for Retired Professors at UFBA (PROPAP/UFBA)^(1)^.

In January 2000, after a public competition, she resumed her relationship with UFBA as Full Professor at the School of Nursing. In 2014, she retired and joined the Special Program for the Participation of Retired Professors in Undergraduate and Graduate Teaching, Research and Extension activities at UFBA, which, at the time of this research, remains active. It is worth noting that, in 2017, she received the title of Professor Emeritus at UFBA^(1)^.

In this regard, the study is justified in unveiling her life, her contributions to the history of HUPES, EEUFBA, from the perspective of analyzing institutional history, in addition to its implications for health, in care and knowledge production in the thematic psychiatry and mental health fields. Furthermore, it is also related to the originality of the study, in the expression of Josicélia Dumêt’s work in psychiatric and mental health nursing, and the science of Brazilian nursing, since she was a prominent researcher.

## OBJECTIVES

To analyze nurse Josicélia Dumêt Fernandes’ life story, with emphasis on her work in the psychiatry and mental health fields.

## METHODS

### Ethical aspects

This research is part of a matrix project entitled “*Modelos de Gestão em Enfermagem: memórias de enfermeiros*”, approved by the Research Ethics Committee. The ethical aspects contained in Resolution 466/12 of the Brazilian National Health Council, which deals with guidelines and regulatory standards for research involving human beings, and in Circular Letter 2/2021 of the Brazilian National Research Ethics Commission (CONEP - *Comissão Nacional de Ética em Pesquisa*), in relation to guidelines for research procedures at any stage in a virtual environment, were respected. The interview only took place with the interviewee’s authorization and written signature of an Informed Consent Form by all participants, and, after transcription, it was validated by her, and only then transcribed for production and analysis of results. The process was guided by the COnsolidated criteria for REporting Qualitative research (COREQ), in the Brazilian Portuguese version, composed of criteria for reporting qualitative research^(2-4)^.

### Theoretical-methodological framework

Foucault’s philosophical framework was used, which addresses the constitution of subjects, pointing to the theoretical aspects of self-techniques, divided into four main groups: (1) production techniques, which allow producing, transforming or manipulating things; (2) sign system techniques, which allow the use of signs, meanings, symbols or meaning; (3) power techniques, which determine individuals’ conduct and subject them to certain ends or domination, objectifying the subject; (4) self techniques, which allow individuals to carry out, with their own means or with the help of others, a certain number of operations on their own bodies, souls, thoughts, conduct and way of being, in order to transform them with the aim of achieving a certain state of happiness, purity, wisdom, perfection or immortality^(5)^.

### Study design

This is historical, qualitative research, with a biographical focus on nurse and professor Josicélia Dumêt Fernandes’ professional life, in the psychiatry and mental health fields. In the context of qualitative research, this type of approach, the life story, allows to explore, discover and assess how people, through their experiences, understand their past, link it in the social context and give meaning to it in the present moment^(6)^.

### Data collection and organization

In order to carry out a historical reconstruction of the biographer, semi-structured interviews, for the production of an oral source, and documentary research were used as data collection techniques. An interview was carried out virtually via the online platform Google Meet^*®*^, lasting around 46 minutes, conducted by two researchers who were the authors of this manuscript. Initial contact with the participant took place by phone and email.

Data was collected from September to October 2021. Information was also collected from the Holder’s Memorial, provided by the professor herself, and from the *Curriculum Lattes*.

The statements from the interview are identified by the letters “INT”, followed by the initials of the professor’s name “JDF”. Regarding the excerpts extracted from documentary collection, they are represented by the letters “DOC”, followed by the initials “JDF”.

### Data analysis

For data analysis, we opted for the content analysis method^(7)^, which included the following steps: clipping of recording units; frequency-based enumeration; treatment of results; and interpretations. Data categorization was based on recording units and manifest content, making it possible to identify, throughout the interviewee’s life, moments that connect with her trajectory in the psychiatry and mental health fields. At this stage, the possibilities of categories were compared with Foucault’s philosophical framework, in an analysis framework, allowing the finalization of the categories. In the data organization stage, qualitative data analysis software (WebQDA^®^) was used to support data analysis.

## RESULTS

We presented and discussed the results of this study, anchored in Foucault’s theoretical-philosophical framework, emerging four categories that are willing to follow.

### Transforming herself and mental health practices

Nurse and professor Josicélia Dumêt Fernandes conducted her professional practice through transformations of thoughts and conduct in the face of constant reflection on the reality that surrounded her, which made it possible to overcome stereotypes, adopt theoretical frameworks and new approaches in the practice of care and teaching in mental health:

*Contained within the boundaries of my training, I looked with sympathy at transformative movements in the professional field. I sought to break the bonds and overcome certain contradictions, realizing the narrow limits imposed by dominant nursing teaching and practices and, particularly, by mental health practices.* (DOC-JDF)

*Little by little, I followed the reforming and renewing movements in the mental health field in Bahia and, gradually, I established an identity with them, even in the university area and, particularly, in the psychiatry field. I participated in critical discussion processes in Bahian nursing. I sought a new conception for my practice. I felt the need to question it in order to (re)construct it, through ruptures in what was established. To achieve this, it was necessary to enter into a process of building a new framework that had as its axis the dialectical movement of life, seeking a new conception of health practices and, more specifically, nursing.* (DOC-JDF)

*I began to realize, with a little more clarity, that my practice was circumscribed and limited as well as covered by the repressive and custodial characteristics of psychiatry. This perception made me adopt an awareness-raising stance, i.e., at the time I was caring for a patient, I tried to clarify for him and his family why the situation they found themselves in and what they could do to overcome it. Likewise, together with the students, I tried to clarify for them the need for a more humane and fairer practice.* (DOC-JDF)

This process of transformation of herself and her practices is shown continuously in the nurse and professor’s history.

*In 1988, I left the school management and, in 1990, I retired. I retired, but I continued developing; I didn’t stop my activities.* (INT-JDF)

*In 2014, I retired. I turned 70; in public service, anyone who turns 70 has to leave. Although I was at the peak of my productivity, at the time, I was the graduate professor with the greatest scientific production. I had to leave, physically leave, I had no connection, but I started to develop activities in this cooperation program between retired professors and the university. As I am today, I am still a graduate professor, I am a project coordinator at CNPq. Since the 2000s, I have coordinated research at CNPq. I am a researcher 1. I continue with guidance activities and collaborating with the School of Nursing.* (INT-JDF)

### (Re)framing professional practice

In her work as a psychiatric nurse at HUPES, Josicélia Fernandes experienced moments of reframing her practice, given the changes that were occurring in the psychiatry field. These transformations began with the weakening of conceptions of care, based on isolation and repression, which underpinned nursing practices, as observed in the speech:

*With the implementation of these community psychiatry movements, we began to put aside that psychiatry of custody, isolation, repression, and began to have some modernized guidelines, i.e., we began to operate with open doors.* [...] *It was a very difficult move for us to stop having bars, but the hospital didn’t allow us to not have bars; the balconies were all with railings. But, on the other hand, we left the door open, but it was a controlled open door, there was a nursing staff at the open door, it was an open door to say it was open.* (INT-JDF)

Furthermore, she used courage as an important symbol in the construction of an objective: selection for the EEUFBA assistant professor competition.

[...] *it made me decide to take part in the School of Nursing competition for the position of assistant professor in the subject of Psychiatric Nursing. My first research work would already be a thesis. How much courage!* [...] *I entered a field that was familiar to me* [care for psychiatric patients]*, however the method for walking in this field* [the scientific method and research methodology] *was, until then, unknown to me. But, as I said, there was a lot of courage* [...] *and I began to discover what was unfamiliar to me - the research method. Along this path, I prepared the thesis “Nursing care in the face of anxiety triggered by neuroleptic therapy”, presented to the School of Nursing and approved for the Assistant Professor competition at that teaching unit.* (DOC-JDF)

### Nursing practice and power relations

Josicélia Fernandes’ experience also made it possible to point out the direction of nursing care at the time, which was centered on traditional treatment, with a focus on the medication approach, insulin therapy and electroshock. It is worth highlighting the difficulties she faced in managing the nursing service for the necessary changes in nursing care, as the organization was structured around a controlling and punitive care model, as shown in the following statements:

*It was very difficult to manage at that time, because the hospital was still designed with an understanding of repressive psychiatry. The psychiatry of electroshock and insulin therapy. So, it was very difficult for us to break with this paradigm and start more modernized psychiatric care, in keeping with the times. I remember there were episodes that were very stressful, it was very difficult. Not today, today things are much lighter* [...] *I would say much lighter, today the understanding of psychiatric care is very different from what it was 10, 20 years ago.* (INT-JDF)

[...] *the ward operated in accordance with the characteristics of psychiatric care at the time. It was drug treatment, there was no occupational therapy, no activity other than the patients staying in bed. And they spent 24 hours there in the ward receiving medication. Therapeutics at the time, in addition to chemotherapy, also included electroshock - electroshock therapy and insulin therapy. This was the treatment regimen at the time.* (INT-JDF)

The results also show that the nurse stood out, as demonstrated by the invitation she received from HUPES management to be a nurse and head of nursing in the psychiatry ward.

[...] *I was no longer a nurse on duty, I was invited to be the head of nursing at the psychiatric unit at what was called Hospital Edgard Santos at the time. And then, as I already had this background from working in a psychiatry field, I went to head the psychiatric unit.* (INT-JDF)

Finally, this category also indicates the relationship between the nurse and professor with influential professionals in the health and teaching areas, who helped in their professional practice:

*In facing this challenge, I counted on the remarkable collaboration of Professors Maria Hélia de Almeida and Therezinha Teixeira Vieira to support activities related to the administration of nursing services and nursing care for clinical-surgical patients. This support was very valuable, considering that many of the patients admitted there had not only psychiatric problems, but general problems* [...]. (DOC-JDF)

*The encouragement from Professor Stela Santos Sena made me decide to take part in the School of Nursing competition for the position of assistant professor in Psychiatric Nursing. With the help of this professor and Professors Maria Hélia de Almeida and Maria Ivete Ribeiro de Oliveira, I faced the challenge.* (DOC-JDF)

### The paths and implications in the psychiatry and mental health fields

Josicélia Dumêt Fernandes, before starting her academic professional life, worked at a psychiatric hospital in Bahia, at the time, considered, for the historical moment, an innovative organization, as evidenced in the following statements:

*The following year* [1966]*, already graduated in nursing, I participated in the preparation of the draft regulations for that hospital and became Head of the Nursing Service. For me, it was a great challenge to occupy a management position as a recently graduated professional.* (DOC-JDF)

*The experience at Casa de Saúde Ana Nery was a rewarding challenge. Through a technical and social relationship with the other work team members, she was able to implement an experience, hitherto unprecedented in Salvador, that of the “nursing secretary”. This experience favored the possibility of the nurse not being tied to bureaucratic activities, being able to provide direct patient care, which was not common at the time.* (DOC-JDF)

She also worked at *Hospital Professor Edgard Santos*, known as *Hospital das Clínicas*, currently called HUPES, where she was invited to take on the position of nurse on duty in the psychiatric ward. At the same time, she began to participate in the Teaching Care Integration Program, in which she developed teaching activities at EEUFBA, and took over the psychiatry ward coordination.

*When the hospital management invited me to be a hospital nurse, I immediately accepted, because I no longer had the same charm I initially had with the hospital I was working at. I accepted and started my activities, I started working as an afternoon and night nurse; At the time, she was called a nurse on duty.* (INT-JDF)

*As I already had an activity in the psychiatry field and the School of Nursing had one at the time, I developed a Teaching Care Integration Program* [CIP]. *Nurses who worked in a specific area of activity collaborated with the teaching of the School of Nursing in that specific area. So, I was invited, I was no longer a nurse on duty, I was invited to be the head of nursing at the psychiatric unit at what was called, at the time, Hospital Edgard Santos.* (INT-JDF)

Among her activities, during the period in which she headed the psychiatric ward, Josicélia Dumêt Fernandes highlighted her participation in the Day Hospital service implementation and in the coordination of therapeutic activities with users and families.

*As head of nursing at the psychiatric unit, I implemented and coordinated the “Day Hospital” care modality - an intermediate care modality between outpatient care and full hospitalization -in addition to participating intensely in the “Therapeutic Community” activities, where I coordinated groups of hospitalized patients, former patients and family members.* (DOC-JDF)

In 1970, the nurse and professor took the exam to enter UFBA and, in 1971, she became part of the School of Nursing permanent staff, maintaining her activities at the university hospital until 1984.

*In October 1970, I was qualified, through this competition, to occupy the position of assistant professor for the subject of Psychiatric Nursing. In February 1971, I became part of the Universidade Federal da Bahia School of Nursing permanent staff* [...]. (DOC-JDF)

*In addition to being a professor, I headed the psychiatric nursing unit. In the morning, I carried out activities at the hospital, psychiatry activities, and in the afternoon, I was at the School of Nursing teaching classes preparing student orientation, research, participation in administrative activities.* (INT-JDF)

[...] *in 1984, I stopped heading the unit to become director of the School of Nursing. I was the first director elected by the community* [...]. (INT-JDF)

However, it is important to note that, during her career, she was always attentive to political aspects and their implications. Still at the academy, as a nursing student, she was attentive to discussions on the new curriculum and participated in the movements of UFBA and the Brazilian Nursing Association (ABEn - *Associação Brasileira de Enfermagem*).

*I was in the second year of the course when I noticed the movement of the School of Nursing and the Brazilian Nursing Association/Bahia Section* [ABEn/Ba] *who, immediately, were dissatisfied with the new curriculum, where the view of man as a being predominated. biological, masking the social aspect of the health/illness process. As a student, I then began to participate in the school and ABEn/Ba movements, contrary to the approved curriculum. I engaged in the discussions, in order to explain to my colleagues the contradictions contained in Opinion 271/62 so that we could collectively find strategies for appropriate solutions that are in line with the interests of the category.* (DOC-JDF)

In her work as a professor and researcher at EEUFBA, in 1994, she contributed to the creation of the Mental Health Study and Research Group (GESAM):

*In 1994, together with Professor Maria Rita Oliveira, we took the first steps towards the implementation of the Mental Health Study and Research Group* [GESAM]. *This group, linked to psychiatric nursing and mental health of the EEUFBA Undergraduate Course, had multi and interdisciplinary characteristics, favoring the development of integrated research, thus overcoming the fractionation of knowledge into isolated branches.* [...]. (DOC-JDF)

She also developed graduate activities at EEUFBA. She started with classes on psychiatry and, throughout her career in the program, developed dozens of orientations for master’s and doctoral students.

*I began my activities with the school’s graduate course, teaching classes on nursing aspects in psychiatric therapies in the specialization course in medical-surgical nursing, in the form of a residency, taking into account the theme of medical-surgical nursing III. Regarding my participation as a professor in extension, updating and training courses, classes on sexual orientation during pregnancy stand out: psychological aspects, application of the scientific principles of psychiatric nursing in the health care reality of Salvador, psychiatric emergencies and nurse/patient relationship. Of these courses, I was a professor and coordinator of the last three.* (DOC-JDF)

[...] *I carried out academic guidance activities, participation in examination boards of doctoral and master’s courses and in selection processes.* (DOC-JDF)

Dr. Josicélia Dumêt Fernandes’ experiences, both as a nurse at the university hospital and as a professor and researcher at the university, boosted her name as a state and national reference in the mental health field, notably by providing consultancy services for health organizations, universities, scientific journals, in addition to numerous lectures at national and international scientific events.

*To this day, I am a consultant, reviewer of magazine articles, from several Brazilian, national and international journals, always in the mental health nursing field and also in the education field* [...]. (INT-JDF)

*Due to the work developed in the technical team of the therapeutic community of the Psychiatric Clinic of Hospital Professor Edgard Santos, I started to be invited, mainly, by the Brazilian Nursing Association/Bahia Section, to give lectures and conferences on topics related to nursing in the therapeutic community and nursing. in a psychiatric clinic at a General Hospital.* (DOC-JDF)

*At a national level, I was invited to participate in several scientific events, giving lectures and conferences on topics related to the training of human resources in nursing and psychiatric nursing.* (DOC-JDF)

## DISCUSSION

The transformation of oneself and mental health practices is intertwined in Josicélia Dumêt Fernandes’ story. The professor and nurse’s constructive questions went through transformations experienced in her personal and professional trajectory, always in search of breaking paradigms involving nursing training, care and management in the psychiatry and mental health fields.

These events express the governability of the self, which implies one’s relationship with oneself, in a set of practices through which it is possible to constitute, define, organize and instrumentalize the strategies of individuals in their freedom. Individuals try to control, determine and limit the freedom of others and, to do so, they have certain instruments to govern others. This is based, then, on freedom, on the relationship between oneself and the other^(8)^.

It is evident that she is a political subject, whose practice refers to the sphere of freedom, thought and human action, inherent to the encounter, which is expressed in individuals’ private and social life, allowing the adoption of a critical, reflective and aware stance towards reality, with striking implications in her professional category^(9)^.

The 1960s were marked by changes in the way of thinking and practicing psychiatry, in addition to advances in the institution of public policies in mental health. In 1966, the strategy used was the Health Campaigns, which shifted the centrality of the medical clinic towards community action in public health. Still with remnants of asylum care, this experience was influenced by the innovations of European psychiatry that were disseminated in Brazil^(10)^.

She demonstrated experiences of mental health practice consistent with the beginning of the process of knowledge transformation in the area. Reframing and symbols of persistence and courage were used in the face of the process of weakening conceptions of care, based on the isolation and repression that underpinned nursing practices until then^(11)^. Even in a psychiatric environment, in which she was more exposed to physical and psychological stress, which made resilience a constant challenge, she reframed her experiences and continued to contribute to mental health care quality.

At another point in this analysis, it is necessary to discuss power as a device for the (re)production of subjects, this understood here as a social practice and, as such, historically constituted, expressed in a set of relationships that are permanently exercised, radiating from bottom to top, like a network that permeates the entire social body^(12)^.

For Foucault, power has a productive effectiveness, a strategic richness, a positivity that targets the human body, aiming to improve and train it. What matters is managing people’s lives, controlling their actions so that it is possible and viable to use them to the maximum, increasing its economic utility, reducing inconveniences, political dangers, increasing economic strength and decreasing political strength. There is no neutral knowledge; all knowledge is political because it has its genesis in power relations^(12)^.

This reframe will be situated with the power relations found in nursing practice. It is worth highlighting that the results indicate that Josicélia Dumêt’s practices, although they were in a traditional psychiatry context, had elements that were close to the Psychiatric Reform discussions that were taking place in the world, but the organization was structured in a still controlling care model.

In this regard, it is necessary to highlight that, historically, the psychiatric hospital is a place of diagnosis, classification and surveillance. She still exercised psychiatric power over the sick to the extent that she exclusively directed the practices with patients. From the 19^th^ century onwards, many of the procedures practiced in asylums were characterized as instruments of coercion aimed at isolation and discipline. Although the hospital where the nurse worked was not psychiatric, it was under the auspices of a dominant care model at the time^(13)^.

For Foucault^(14)^, in psychiatric institutions, the micromechanics of power, based on exclusion mechanisms, surveillance strategies and medicalization of madness, was constituted and exercised in subtle mechanisms that organize and shape a certain knowledge that puts itself into social displacement^(14)^.

Therefore, power relations permeate and permeate health processes and, more specifically, treatment of mental health users. Clearly, standardization of mental health can be perceived by the various interventions and approaches used with these users throughout history and highlighted in the results, in nurse Josicélia’s challenging attempt to align with the practice of overcoming established care paradigms in psychiatry^(15)^.

The results also showed that Josicélia Fernandes had specific knowledge that led her to management positions as well as relationships with influential people. In view of this, it is necessary to point out that the decades of these achievements were permeated by power relations that were based on male chauvinism, with a predominance of male professionals, and the hegemony of the medical category in management positions in health organizations^(16)^.

When situating an archaeological analysis, it can be understood that both discourses and practices express knowledge. When analyzing the professor’s life trajectory, the discontinuous layers of discourse can be vertically excavated, in order to bring to light fragments of perhaps forgotten ideas, concepts and discourses. Archaeological analysis is a description of discourse in search of regularities that function, such as laws that govern the dispersions of utterances that make up that discourse^(5)^.

Therefore, even though they are not explicit in the results, the strategies used by the nurse and professor in acquiring technical knowledge, in the concessions and challenges they faced in these spaces of power, must be considered. But, at the same time, her relationship with influential nursing professionals of the time demonstrates assistance in her professional career, also constituting power relations.

It is also worth highlighting the techniques of production and transformation of subjects, expressed in discursive statements, permeated with characteristics of Professor Josicélia Dumêt’s persona, such as courage, ethical-political commitment to nursing and the Health and Psychiatric Reform, willingness to change and leadership. What is worth highlighting, even though it is not the focus of this study, in addition to the areas discussed here of her work in leadership spaces, Josicélia was also deputy coordinator in nursing of the Coordination for the Improvement of Higher Education Personnel (CAPES - *Coordenação de Aperfeiçoamento de Pessoal de Nível Superior*) from 2008-2011 and had a notable performance at ABEn.

Furthermore, these aspects form a powerful life story, based on the representation for nurses, and, in turn, the implications for collective and individual trajectories in this field of knowledge, giving visibility to elements of the story and place of participation for nurses in construction of new health care perspectives.

Professor Josicélia’s life reveals a trajectory of productions and contributions in the psychiatry and mental health fields. Still in the 1960s, she began her professional life in a psychiatric hospital; she worked for almost three decades in the HUPES psychiatric ward as an on-duty nurse and head of the service; she was qualified as a psychiatry professor at EEUFBA; together with other colleagues in the field, she founded a study and research group in the mental health field; she participated in advisory services for several organizations in the area; she gave numerous lectures; She worked at CAPES, ABEn and had several scientific publications in the psychiatry and mental health fields.

It is also worth highlighting the implementation and coordination of unprecedented services in the field. This trajectory allows us to point out that she dedicated a large part of her life to contributions in the mental health field, which ensured that she was a reference in this thematic field. In this regard, it is noteworthy that the knowledge produced fostered constructions about nursing training and care in the psychiatry and mental health fields, which strengthened her professional identity as a nurse and researcher.

Josicélia Fernandes’ trajectory is crossed by political, social and cultural issues that interfered with mental health care in Brazil. Study states that Brazilian nursing played an important role in transforming care models in the psychiatry field, collaborating with the Brazilian Psychiatric Reform in changing the style of care for people in mental distress^(17)^.

Still from a Foucauldian perspective, one can observe the games of truth, the curves of visibility, the lines of force and escape of this life story, crossings of a dominant logic and practices of liberation. The symbolic struggle between the dominant and hegemonic modes and models of care and the innovative, democratic, humanitarian and participatory models of care stood out^(18)^.

This implies mentioning that Josicélia Dumêt, over the years, contributed to improvements in nurse training, work and provision of care, even though, at the time she worked, Psychiatric Reform and mental health legislation principles in Brazil had not acquired achievements and notoriety. Furthermore, as a member of ABEn, she has been fighting for improvements for the category since its formation. This is a space that has its axis in the defense and consolidation of nursing work, i.e., the nurse was a strategic figure in strengthening the profession in this field^(19)^.

It is important to demarcate the need for the story of a nurse like Josicélia Dumêt, with her contributions to the institutions and the area in which she worked, to be written to make her legacy evident in the historical construction of nursing as a profession, specifically in mental health, thus contributing to the construction of nursing identity and being able to be a source for new research related to nursing history^(20)^.

It is possible to identify, based on techniques for understanding the subject: the power of production techniques, in which the professor produced and transformed her practices and also that of a collective; techniques of sign systems, which allowed Josicélia, through the use of signs, meanings and symbols, to reframe her practices; power techniques, which determined their conduct and leadership capacity, objectifying the subject; and, finally, self-techniques, which allowed the nurse to carry out, alone or with the help of others, a certain number of operations on her body and soul, her thoughts, her conduct, her ways of being, of transforming herself. whether in order to meet a certain state of happiness, of purity, of wisdom, of perfection, or of immortality^(21)^.

### Study limitations

The limitations of this study are related to the focus of this research in one area and the professor’s performance, not totaling all of Josicélia Dumêt Fernandes’ dedication to the thematic area highlighted over these years, in addition to the fact that no people from around Professor Josicélia were interviewed.

### Contributions to nursing, health or public policy

They made it possible to highlight professional Josicélia Dumêt Fernandes’ trajectory as a nurse and professor at the School of Nursing at UFBA in psychiatry and mental health, showing her legacy, based on the transformation and consolidation of knowledge, in conquering leadership spaces in the services in which she worked, highlighted in HUPES and EEUFBA, and in the implementation of services considered innovative at the time.

In this way, this study contributes to the preservation of nurses’ memory and its intertwining with Psychiatric Reform’s history in Bahia in the various dimensions of nursing work, such as care, management, teaching, research and political participation. Likewise, it strengthens the construction of knowledge of nursing discipline and professional identity.

## FINAL CONSIDERATIONS

This study allowed the analysis of Josicélia Dumêt Fernandes’ life story, with an emphasis on the theme and her work in psychiatry and mental health, demonstrating a trajectory outlined by the search for transformation of self and mental health practices on an ongoing basis, seeking to break paradigms in the psychiatry and mental health fields. The existence of power relations in her professional practice was also highlighted, experiencing challenges due to having elements in her practice that were close to Psychiatric Reform discussions in the face of a repressive psychiatric care model.

The professor experienced the beginning of the process of transformation of mental health care, needing to reframe her practice, with a trajectory marked by productions and contributions in psychiatry and mental health. She worked in management and assistance positions, was a professor and researcher in the area, implemented innovative services, dedicating a large part of her professional career to mental health.

The study of the professional’s biography shows her legacy in psychiatry and mental health, but also reveals her lucidity, commitment and persistence in strengthening the nursing field. She continues to enable and support her practices in nursing evolution and transformation, being a reference in the training of several generations of nurses.
